# Supplementary material for: Gene Targeting to the Cerebral Cortex Following Intranasal Administration of Polyplexes
Source: Pharmaceutics. 2022 May 26;14(6):1136. doi: 10.3390/pharmaceutics14061136 (PMC9231247; doi:10.3390/pharmaceutics14061136)
Supplement: Supplementary file 1 [file pharmaceutics-14-01136-s001.zip › pharmaceutics-1664801-supplementary.pdf]

**Supplementary Information**

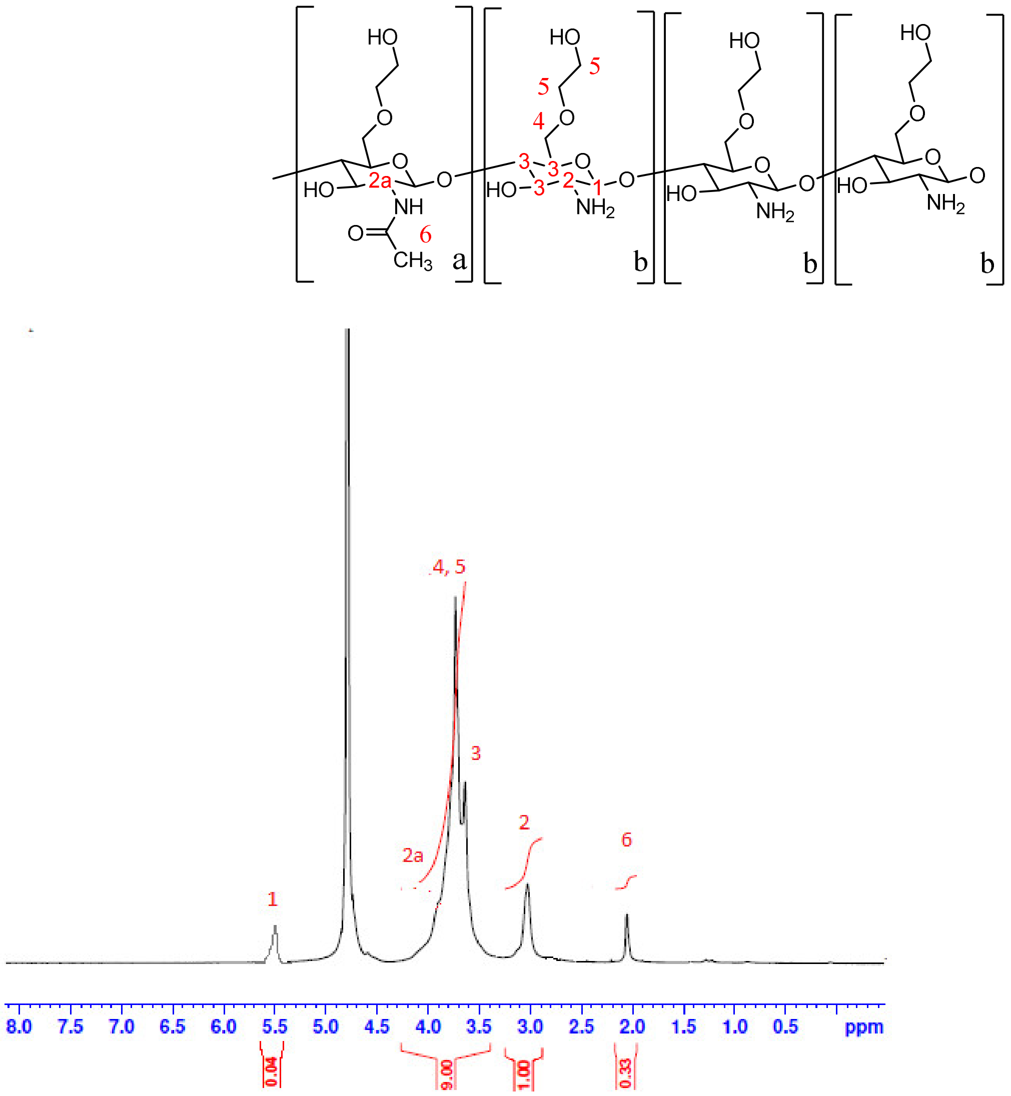


**Figure S1:** Degraded GC37 $^{1}$H-NMR ($D_2O$): $\delta_{2.1}$=[$CH_3$-CO-NH-], $\delta_{2.9\text{-}3.2}$=[-CH-(OH)-CH-($NH_2$)], $\delta_{3.4\text{-}4.2}$=[-CH(OH), -$CH_2$(OH) and -CH-(OH)-CH-NH-CO-)], $\delta_{4.79}$=[$D_2O$], $\delta_{5.5}$=[-O-CH-O-].

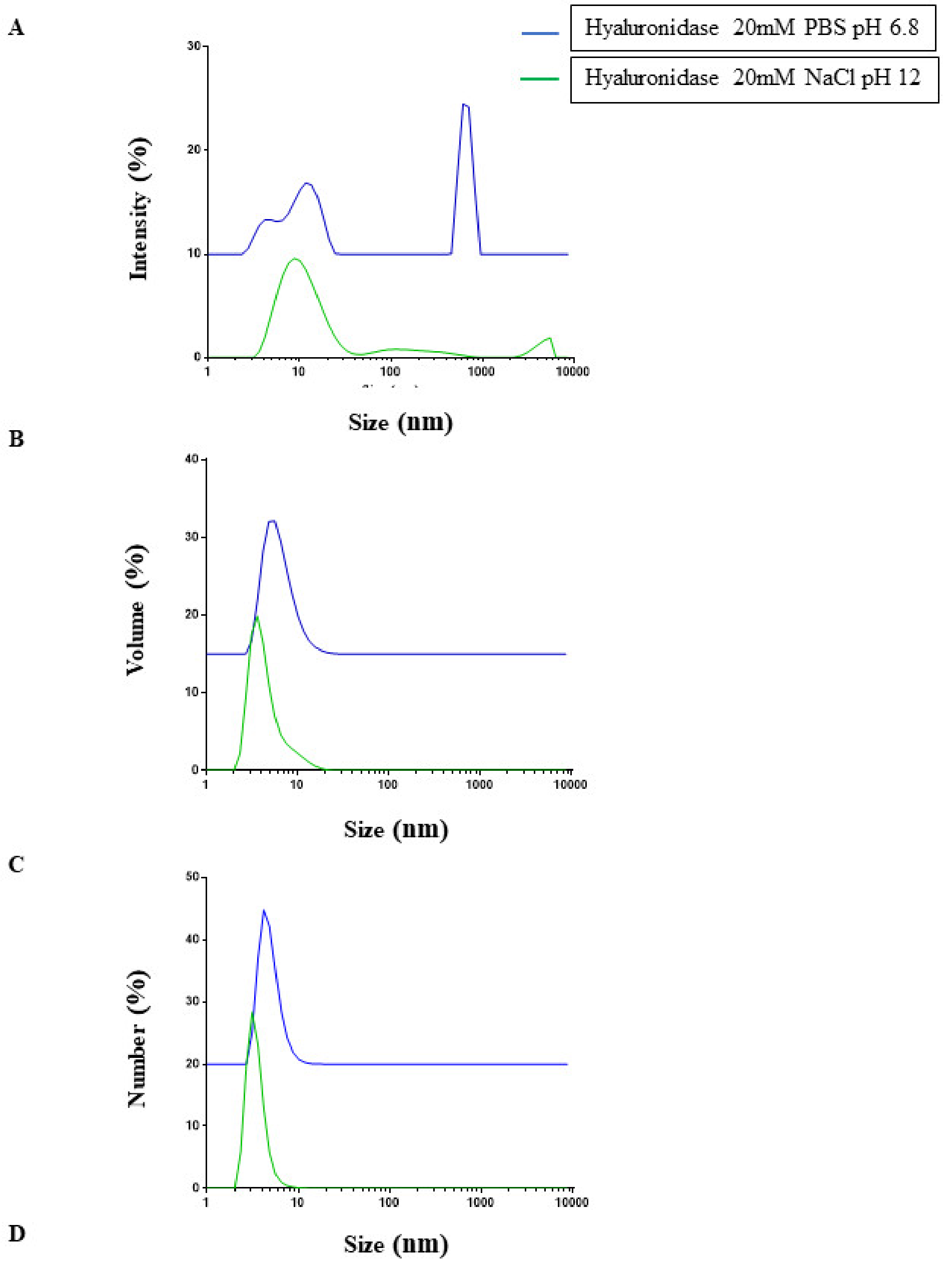


D

| Hyaluronidase | Size (nm) | PDI | Zeta potential (mV) |
|---|---|---|---|
| 20mM PBS, pH= 6.8 | 4.6 (14%) 11 (42%), 700 (44%) | 0.280 ± 0.076 | -4.5 ± 1.2 |
| 20mM NaCl, pH=12 | 11 (82%), 206 (11%) | 0.541 ± 0.096 | -26 ± 3.1 |

**Figure S2:** Size distribution plots of hyaluronidase by intensity (**A**), volume (**B**) and number (**C**) in 20 mM PBS, pH=6.8 (blue plots) and 20 mM NaCl pH=12 (green plots). (**D**) Size, PDI and zeta potential of hyaluronidase in 20mM PBS, pH=6.8 and 10mM NaCl, pH=12. The data is representative of three independent experiments (Mean ± SD, n=3)

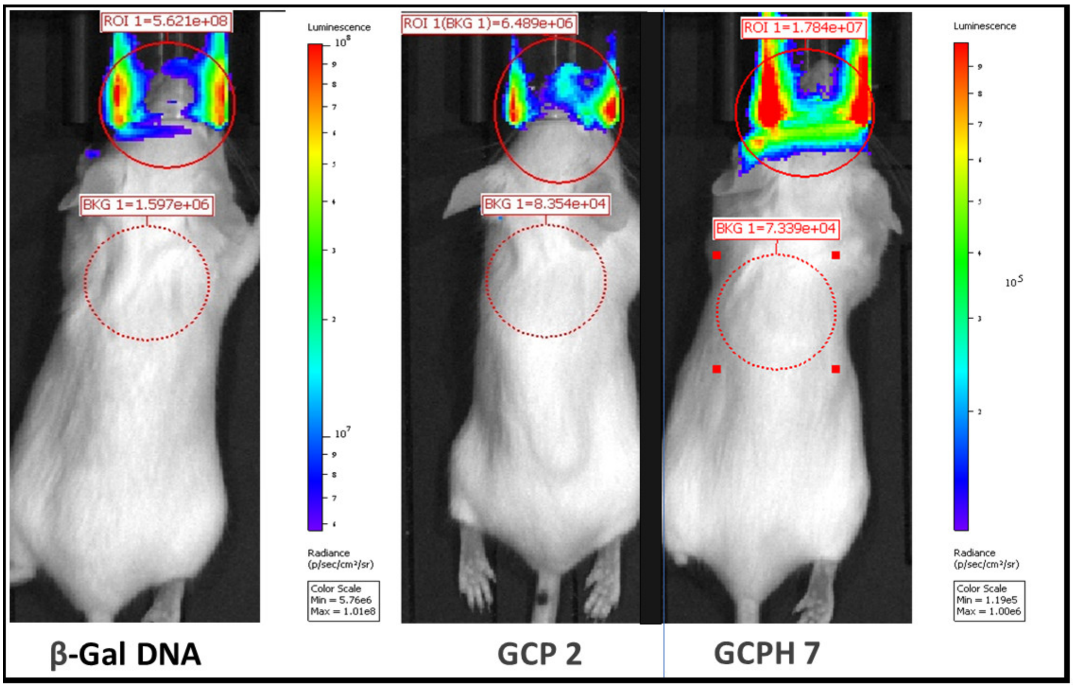


**Figure S3**. Representative IVIS images 24 h post intranasal administration of polyplexes and 15 mins post intranasal administration of the substrate at β-Gal DNA concentration of 84 μg mL $^{-1}$ or 0.067 mg kg $^{-1}$. The signal intensity is very strong and so it appears to bleed out from the animals for the exposure time of 240s.
